# Organizational Development as Generative Entrenchment

**DOI:** 10.3390/e24070879

**Published:** 2022-06-26

**Authors:** Cody Moser, Paul E. Smaldino

**Affiliations:** 1 Department of Cognitive and Information Science, University of California, Merced, CA 95343, USA; 2 Center for Advanced Study in Behavioral Sciences, Stanford University, Stanford, CA 94305, USA

**Keywords:** organizations, generative entrenchment, cultural evolution, information constraints, group selection

## Abstract

A critical task for organizations is how to best structure themselves to efficiently allocate information and resources to individuals tasked with solving sub-components of the organization’s central problems. Despite this criticality, the processes by which organizational structures form remain largely opaque within organizational theory, with most approaches focused on how structure is influenced by individual managerial heuristics, normative cultural perceptions, and trial-and-error. Here, we propose that a broad understanding of organizational formation can be aided by appealing to generative entrenchment, a theory from developmental biology that helps explain why phylogenetically diverse animals appear similar as embryos. Drawing inferences from generative entrenchment and applying it to organizational differentiation, we argue that the reason many organizations appear structurally similar is due to core informational restraints on individual actors beginning at the top and descending to the bottom of these informational hierarchies, which reinforces these structures via feedback between separate levels. We further argue that such processes can lead to the emergence of a variety of group-level traits, an important but undertheorized class of phenomena in cultural evolution.

## 1. Introduction

Within cohesive, collaborative societies, humans are subdivided by a diverse variety of interests and goals. When interests and goals align between individuals or groups and the need for coordinated action is apparent, an organization is formed. Hall [1] defines an organization as “a collectivity with a relatively identifiable boundary, a normative order, ranks of authority, a communications system, and membership coordinating systems”, and we adopt this definition as adequate for our purposes. Central to an organization is the formalization of both roles and goals [2]. Just as the goals of individual humans differ, so too do the goals of their organizations. Organizations are also dynamically shaped by their real and perceived functions [3,4]. An organization that serves to bring a product to the market and sell it to consumers is different from an organization that sells advertising or those that distribute food to the homeless, confer advanced degrees to students, or organize mass protests.

As a method of structuring assemblages of agents to align with large-scale goals, a critical task for an organization is how to most efficiently structure hierarchies to direct both information and resources to actors tasked with solving sub-components of the broader problem [5,6]. As noted by Simon [6], “organization behavior is a complex network of decisional processes... The anatomy of the organization is to be found in the distribution and allocation of decision-making functions”. Such “optimizing” approaches to understanding organizational formation help to explain why organizations may generally take some concrete structure, but they do not explain the processes by which any particular structures emerge, especially in the absence of strong managerial foresight, an absence that is probably quite common [7,8,9,10,11]. At the same time, structural similarities between organizations indicate that organizations really are optimized for efficient decision making, with organizational structures clustering around two poles representing hierarchical, pyramidal structures at one end and flat structures with only loosely formalized subcomponents at the other [12,13]. These two attractor-like poles of organizational structure are often noted with respect to the arrangements of what we call “formal” organizations present in industrial society, but similar patterns have been identified in cross-cultural examinations of human organization in non-industrial society [14,15]. It is still not well understood why certain structures and not others emerge and what combinations of top-down and bottom-up processes lead to these commonalities. With the study of organizational structure often focusing on mature organizations, the developmental processes leading to the formation of structure have been left in relative opacity [16,17].

Why organizations look similar may reflect similarities among the decisions, conscious or unconscious, made by founders and managers in the process of organizational development. These include managerial heuristics, cultural perceptions of how organizations “ought” to be arranged, and trial-and-error [18]. At the same time, it remains unclear why founders and managers in very different organizational domains should make such similar decisions. A second possible explanation, complementary to the first, is that structural similarities among organizations reflect a form of survivorship bias, such that organizations that look very different are so inefficient that they either change their structure or perish. This evolutionary argument seems plausible. However, a key point known to evolutionary theorists who consider development (the so-called “evo-devo” perspective) is that the strength of selection is not uniform over the course of the lifespan. Rather, early-developing features often have the most substantial influence on evolutionary fitness, leading to more severe constraints (and thus greater uniformity) among even very different organisms early in their developmental history. This process of early mutual shared features between different types of organisms thus helps to constrain their potential morphospace, limiting organizational entropy [19]. A corollary to this idea is that later-developing features will exhibit more variation in their features, meaning that development actually becomes more entropic over the lifespan when comparing across species. In this paper, we explore the narrative that a similar pattern of increasing entropy can be observed in the development of human organizations.

We develop an evo-devo framework for explaining organizational similarity and diversity in terms of the feedback between the units comprising an organization and the organizational structure itself. In addition to explaining organizational structure, this framework contributes to explanations of the emergence of group-level traits in human societies, which are subject to social and cultural evolutionary forces that require explanations distinct from explanations for the evolution of individual-level cultural variants [20]. Luckily, there already exists a systems framework linking the behavior of individual units to the emergence of complex structures, one originally developed for organismal theory in biology: *generative entrenchment*. We argue that the theory of generative entrenchment can provide additional utility as a general theory of hierarchical emergence, especially in the context of organizations.

The remainder of the paper proceeds as follows. We begin by introducing the concept of generative entrenchment and its general principles. We then discuss how the processes that create generative entrenchment may generally apply to both flat and hierarchical organizations during their development and how the general principles of generative entrenchment appear in organizations. Finally, we discuss the role that generative entrenchment plays in the cultural evolution of organizations, with a broader discussion on how organizational structures are transmitted from one organization to others. Intriguingly, initial theories of generative entrenchment had their origins in organizational theory [21,22], so this paper, in a sense, reflects a sort of homecoming.

## 2. Generative Entrenchment

**Figure 1:**
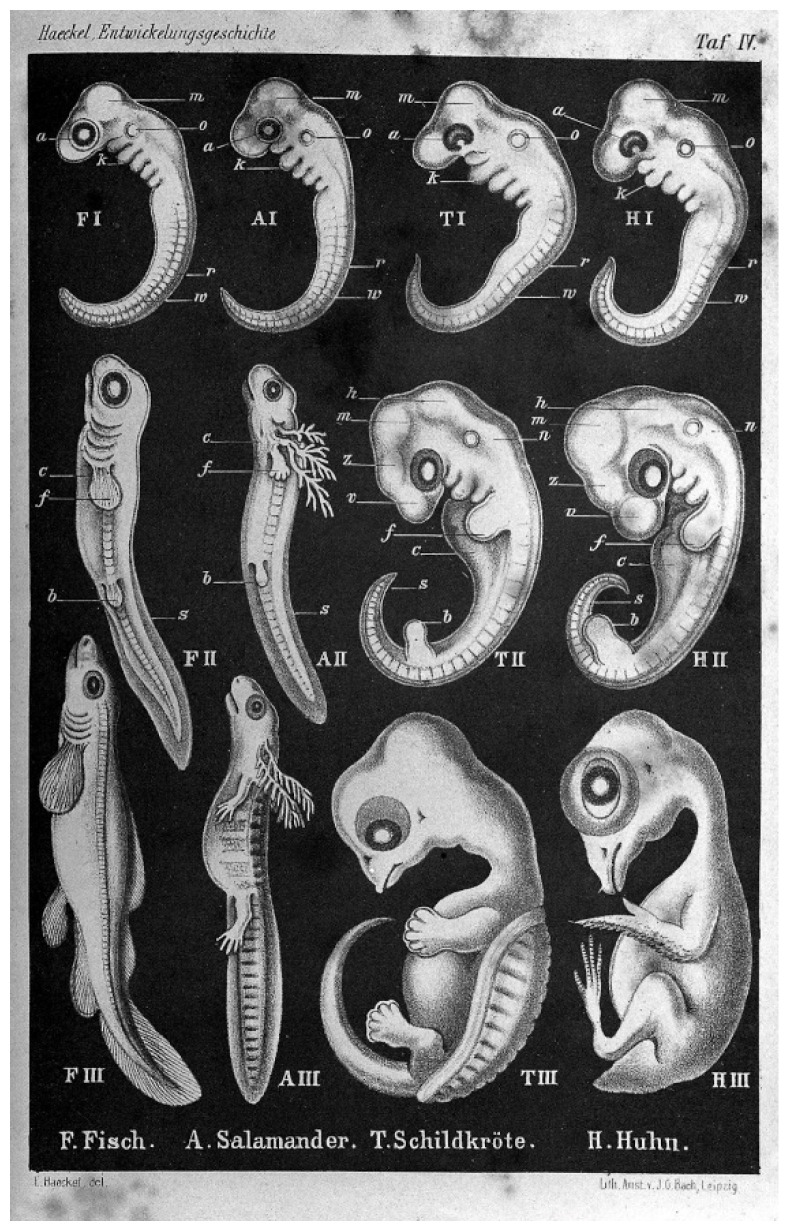
Embryonic drawings by Ernst Haeckel, from his 1874 book, *Anthropogenie*.

Generative entrenchment is a feature of developmental systems in which developmental events possess downstream dependencies (generativity). Earlier-occurring events involve both more numerous and more consequential downstream effects, which is to say they are more entrenched. Generative entrenchment explains why embryos of genetically distal classes of vertebrate animals (be they fish, mammals, birds, reptiles, or amphibians) look strikingly similar in the first days of their development [21,22]. This similarity led the 19th century German biologist and artist Ernst Haeckel to erroneously conclude that each animal revisited earlier evolutionary stages during development (that is, that “ontogeny recapitulates phylogeny”). His illustrations are shown in Figure 1. However, a more plausible explanation is that mutations involving genes that are active during the earliest developmental stages are most likely to have large effects on the foundational characteristics of chordate anatomy and are therefore likely to be deleterious and strongly selected against. Changes involving genes that are active only after the foundational body structure is in place are less likely to be catastrophic, thereby allowing the diversity of vertebrate forms to evolve. We may say that these latter genes are less entrenched.

**Figure 2:**
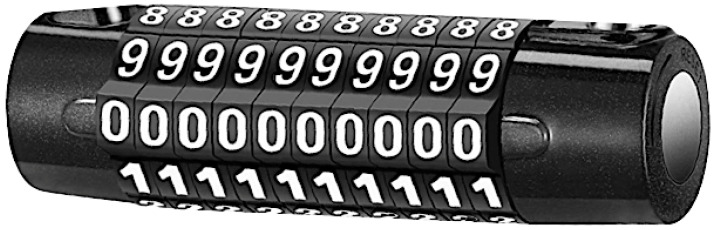
A 10-wheel combination lock.

In his discussion of generative entrenchment, Wimsatt [21,22] appeals to the analogy of the “complex lock” introduced by Herbert Simon [23]. Simon developed this thought experiment to justify the study of complex systems in terms of their individual components. Simon noted that unlocking a combination lock comprised of 10 wheels, each with 10 numbers (Figure 2), is an extremely difficult problem if you do not already know the combination, because a brute-force approach requires guessing blindly among 10^10^ possible combinations. As such, most wheel turns convey close to zero information, with every incorrect combination equivalent to every other one. Simon proposed that progress on thorny complex problems could be made by imagining complex systems instead as locks in which each wheel can be solved independently. If each individual wheel in the lock emits an audible “click” when it is turned to the correct position, the problem of solving the lock is reduced from a blind search among 10^10^ possible combinations to ten simple searches each among only 10 possibilities. In this case, there is much more information to extract from the lock, even if there remains zero *mutual* information shared between each pair of wheels.

Simon proposed that complex systems are often decomposable in this way. However, Wimsatt [21,22] argues that many systems are not so easily decomposed and instead exhibit path dependency, in which decisions made in one stage of the solution process affect the value of decisions made during later stages. They propose that the case of organismal formation is better modeled as a “developmental lock”. This developmental lock looks just like the combination lock in Simon’s thought experiment. Here, however, problem space is not decomposable when solved from right to left, only left to right. This is because a change in the value of any wheel in the lock resets the solution values for all wheels to the right of that wheel but not to the left. Changes to more leftward wheels therefore induce larger increases to the entropy of the system than do changes to the more rightward wheels. In Simon’s terminology, solving the developmental lock becomes easily decomposable only when wheels are solved left to right, allowing one to experiment at each step as if one is solving 10 independent problems. Meanwhile, solving from right to left remains an exercise in futility.

Systems similar to developmental locks, Wimsatt argues, are present in biological development, where early changes in an organism affects later ones. Because structures that develop earlier have more downstream consequences than ones that develop later, these structures ought to be more strongly conserved with regards to mutation and natural selection. As a consequence, there will be less variation in early-developing structures than in later-developing structures, leading to apparently universal similarities in the structures we see in the early embryonic development of vertebrates. Based on the logic of generative entrenchment, Wimsatt [21,22] proposes several principles linking both development and evolution:

1. The proportion and number of mutations that are adaptive declines rapidly at earlier stages in development.
2. Evolution should be increasingly conservative at earlier stages of development (with most adaptive evolution occurring through modifications occurring later in development).

*They further propose that Principles 1 and 2 hold true because:*

3. Features being expressed earlier will have a higher probability of being required for features appearing later.
4. Features expressed earlier will, on average, have a larger number of downstream features dependent on them (i.e., they are more generatively entrenched).

In generatively entrenched systems, initial forms of structure generate further forms of structure, which are themselves dependent on the conditions of the initial structures. This causes complex structures to become entrenched, with generativity happening at all scales of structure, and the highest levels of entrenchment being reserved for the most baseline and earliest developing structures. The path dependency in generative entrenchment is maintained via feedback between cause and effect, where newer, more derived structures must provide feedback to the performance of the whole system for the derived structures to be maintained [24]. The concept of generative entrenchment therefore is a general description of hierarchical and self-assembling path-dependent systems.

## 3. Generative Entrenchment of Organizations

Simon’s [23] analogy of the complex lock was originally used to explain organizations, and only later, it inspired the biological literature on generative entrenchment. However, the idea of generative entrenchment has not yet found its way back into organizational theory. Several authors have discussed the application of generative entrenchment to cultural evolution more broadly [24,25], but they have not explicitly focused on organizations. It is possible, of course, that organizations do not actually exhibit generative entrenchment. However, we believe they do, at least insofar as the categorization allows us to draw meaningful inferences. Like an organism, organizations themselves go through a historical path of development, starting small and growing later, starting general and moving towards specificity, often exhibiting hierarchical dependent organization and often possessing multiple levels of organization [5].

Many cultural niches involve movements or market conditions for which there are heavy demands for largeness, such as the many benefits afforded by economies of scale. Even in these cases, newly emergent organizations tend to at least start small. At the beginning of organizational formation, hierarchy may not be present, even if a managing founder, or a group of founders are. Some organizations opt for “flat” or horizontal structures, in which tasks are dealt with equally and hierarchical delegation is not present [17]. These groups run like traditional teams without a manager present. In a flat structure, the decision-making apparatus is distributed among the employees, which can speed up decision making, as information within the organization does not have to travel up a hierarchy, facing the potential for a veto at each step, before coming back down [12,17,26].

Nonetheless, very few large organizations exhibit a flat structure, and very few, if any, old organizations do. Why? One reason is that such organizations do not tend to survive in this form for long [27,28]. Although flat organizations can respond to market demands relatively quickly, as an organization settles into a niche and begins the process of focusing its resources on exploiting it, the decentralization of administrative control becomes a hindrance to the organization’s ability to focus on the few core tasks that are most important to its operations [29,30]. Several studies have found that organizations with flat structures either exhibit less long-term viability than organizations with a hierarchical structure or later attempt to switch to a hierarchical structure (we intentionally choose the term attempting here because the switch from a flat to hierarchical structure is a difficult one to make, and managerial conflicts in this stage occasionally lead to organizational collapse) [31,32]. Consider the infamous case of id Software, which pioneered the use of 3D computer graphics in the video game industry. Following its foundation in 1991, id went on to revolutionize the gaming industry with Wolfenstein 3D (1992) and Doom (1993), yet just five years later, after the release of Quake (1996), the company faced almost certain collapse when 50% of its founders left the company due to “lack of leadership” [33]. Quake went on to be the last original title that the company developed until its acquisition by ZeniMax Media in 2009. A similar story was related by the employees of the web infrastructure firm CloudFlare, a flat organization which lost one-seventh of its employees overnight just three years after founding, and Zappos, an online shopping retailer who similarly lost 15% of its workforce after announcing it was changing to a flat structure in 2013 (it eventually gave up on a flat structure less than a decade later) [34,35].

With regards to specialization, the most senior roles in an organization (those highest in the hierarchy) are generally the least specialized, with the most specialized units also appearing later in the timeline of organizational development, beginning with general managers and later extending C-Suites to include “functional” managers (chief executive officer, chief financial officer, chief operating officer, chief technology officer, and so on) [36]. Even in the early stages of organizational formation, when responsibilities are more or less equally distributed throughout a small, flat group of partners, it is typically understood that this group will form the core of the organization’s administration once the organization grows [37]. For example, Bill Gates started as a core programmer in the early days of Microsoft and maintained a core position, this time as CEO, when the company expanded. The function of the role which the Gates of 1975 played within Microsoft’s organization was similar to the role played by the Gates of 2000 with regards to his primary task of leading an organization that developed and sold functional computer products. The difference is due to the fact that both the task and the organization became more complex, with specific tasks delegated to specific branches at the periphery of the organizational network and general tasks being consolidated at the core. While the role of the leader in a company is generally understood to be a functional unit within an organization on day one, as the problems that an organization faces increase in complexity, so do the problems of leadership. An administrator’s role then becomes primarily one of delegation: creating and coordinating teams of employees specialized to deal with sub-components of the larger problem at hand.

Finally, the presence of both hierarchy and specialization means that organizations are functionally organized, similar to the functional structure of organisms [38]. Genes that are influential early in an organism’s development build the critical, foundational structure of an organism, which in turn scaffold the development of an organism’s apomorphic traits (the specialized traits that are characteristic of a species). Vertebrates, for example, all possess the same basic axial skeletal structure, upon which different appendages (fins, claws, wings, etc.) can be built. Similarly, core roles within an organizational hierarchy work to scaffold the development of future sub-components of the organization’s structure and work to constrain the potential structural space the organization can take on, thus reducing the organization’s entropy [39]. In developing a theory of organizational differentiation, Blau [5] posited that feedback between coordination of administrators was a primary process for maintaining differentiation in organizations, what Buckley [40] deemed the morphogenic (structure transforming) processes of social formation. Just as at the core of each vertebrate organism, there is an unchanging structure of the backbone and neural crest, in organizations, there is often a stabilizing core structure that persists regardless of the specific functional niche that any organization occupies [4,41].

In sum, there are several parallels between organizational development and organismal development, which suggests that both structural phenomena may exhibit generative entrenchment. Unlike organisms, organizational development is not the product of explicit genetic instructions. While the issues faced by an organism are generally shared by all of the organism’s component cells and organs, issues faced by organizations take place on both the level of the organization and on the level of individual actors [5,9], whose incentives and goals are not always aligned. Furthermore, whereas an organism typically develops only once (from juvenile to adult), organizations are in continual development [42]. In order to better understand how aspects of organizational development may be generatively entrenched, we need to say a bit more about the process of organizational development.

### 3.1. The Development of Hierarchical Organizations

Organizations are typically founded by individuals who face limitations not only in their ability to produce products or actions, but also in their ability to acquire and process information. The size of an organization must ultimately depend on the number of individuals needed to achieve its goals. However, the structure of an organization is ultimately related to the flow of information that simultaneously enables and constrains the organization’s members in their roles within the organization. When an organization is first formed, it may consist primarily of a small number of founders who are able to do all the work required by the scope of the organization’s goals. If there is selection for the organization’s size to increase (through opportunities or needs to increase its influence, profits, or output), the organization will grow. The resulting growth then introduces new problems related to informing and coordinating additional individuals in an increasing number of tasks. The decisions made by the founders, whether consciously or not, in their initial expansion efforts subsequently introduce new constraints on further expansion.

Each individual within an organization contributes to the organization’s overarching aim by working on a local task connected to that aim. Each person also has limitations on their informational bandwidth, determined by their personality, specialty, expertise, and management responsibilities (dependencies) over others. In an organization, most information sharing occurs between members of a team. As the tasks faced by an organization increase in complexity, the individual bandwidth experienced by team members may become strained if that team’s subtasks similarly increase in complexity. Teams face several options to resolve this problem. They can add additional members to their team (thus straining more of the team’s bandwidth, as they have more team members to interface with); they can allocate all or part of their task to a lateral team on the same organizational rung (thus losing the ability to control information on their portion of the problem); they can scale back the problem at a net loss to the organization; or they can allocate the task to a new team below them, allowing delegation of the novel sub-subcomponent to a new team faced with this new set of the problem space, but still controlled by the initial team [12].

The dynamic described above creates the conditions for generative entrenchment. Older teams will tend to have more system-critical problems than newer ones. They will similarly have more downstream dependencies. These dependencies are teams or sub-organizations that risk collapse if their superordinate managers fail at their tasks. Such older, core teams will tend to undergo fewer changes in structure, such that these roles appear stable within and across organizations based on sheer necessity; the constraints on their bandwidth mean that they likely will not grow past their early capacity, and issues of sharing critical information on their portion of the global problem mean they likely will not try to grow horizontally. As a consequence, despite changes to the global structure, during this process, the earliest developmental trajectories within an organization are preserved, while overall diversity increases. This relates to a guiding principle in organizational science, that the most important additions to an organization are the first few recruits, as they establish the norms and workplace cultures that guide the decisions of all those who join later [43,44].

The deductions here are not dissimilar to those inductively theorized in Blau’s [5] paper, “A Formal Theory of Differentiation in Organizations”, which sought to explore the link between organizational growth and organizational differentiation. Based on his empirical research, Blau proposed several generalizations about organizational formation. First, increasing size generates increased structural differentiation at a decelerating rate, meaning that the greatest increase in differentiation comes with the first major size increase. Blau further proposed that within this relationship of increasing size, the subunits of differentiation themselves become differentiated in a parallel manner (i.e., such that branches within a branch are subject to differentiation). Second, differentiation enlarges the organization’s administrative components, such that any given manager’s vertical span of control increases. Similar to our proposal that task complexity relates to organizational size and differentiation, the relationship between task complexity of an organization and the “height” of its structure has been extensively discussed elsewhere [17,45,46,47]. In one illustrative example, Anderson and Warkov [48] found that administrative levels scaled with the number of tasks a hospital was designed to solve, even when controlling for the size of each hospital.

### 3.2. The Development of Non-Hierarchical Organizations

Generativity may reliably produce hierarchy within organizations, but not all organizations are built hierarchically. Some organizations lack a hierarchical structure entirely. Take, for example, the structure of Alcoholics Anonymous, which possesses a lateral structure, where individual local A.A. groups are independent and largely autonomous from surrounding groups. Although there is a “leadership”, it itself is democratic and decentralized in its structure [49]. Similar arrangements are found in other organizations. The arrangements of clandestine cell systems, for example, are comprised of individual “contact rings” and, for many within them, an indeterminate hierarchy [50,51]. Another example is the collection of 14 “autocephalous” Christian Orthodox churches, which are independent but nonetheless in communication with one another, forming a ring-like structure in their collective arrangement [52]. These arrangements, which we deem “lateral” arrangements, optimize their organizational structure by taking a flatter or more ring-like shape, which contrasts with the pyramidal shape of more hierarchically structured organizations.

One possible view of these organizations is that they are produced via generativity, but they are not generatively entrenched in the sense that their individual components can be characterized as being upstream or downstream of one another. Instead, each unit of the organization is on the same level. The formation of such organizations can still be characterized by constraints on leaders. However, in this case, when a new problem arises, it is not viewed as a subcomponent of an existing task (which would benefit from hierarchy) but as a new task on par with existing tasks. Instead of relying on differentiation of roles to solve problems of the same task, they rely on segmentation to solve lateral tasks. In these cases, delegating to a level below one’s own would be an inefficient use of both a leader’s and their subordinate’s bandwidth, so a lateral team is created. Relatedly, teams will rarely support the creation of a new unit above them to manage both themselves and a lateral team; instead, autonomy is preferred. Flat organizations therefore attempt to optimize their structures so that autonomous cliques deal with separate, but similarly scaled problems in alternative environments. Such separations may be explicitly geographical, as is often the case with franchises [53], but they can also be problem-based, as in the flattened “Clustered Web” of the Samsung Corporation, where individual programs within the corporation are given vast amount of oversight and expected to form their own networks with problem-specific contractors, limiting the average number of decision-making steps to three people [54]. The tradeoffs between the choice to grow via horizontal segmentation versus vertical differentiation in organizations has previously been noted [13], and in a seminal theory of organizational differentiation, Burns and Stalker [12] argued that virtually all organizations lie at two ends of a pole, with one end maximizing vertical and one end maximizing horizontal growth. Such organizations have similarly been described as either minimizing their vertical entropy (operationalized as differentiation between levels of hierarchy) or maximizing their horizontal entropy (operationalized as differentiation between groups on the same levels) [55].

Why grow laterally when a team could create two teams below themselves and take on the role of management? The answers to such a question may not necessarily lie in the scope of individual constraints but instead in how organizations are shaped by the unique functional niches within which they are positioned [12]. Some research on organizational structure has maintained that vertical growth is optimized in stable environments [56,57] and where organizations focus on complex tasks [17,46,58]. Thus, horizontal structures may be optimal when environments are highly variable and/or when tasks are relatively simple. This hypothesis finds agreement with formal theories of cultural evolution, indicating that egalitarian social learning, in which individuals adopt behaviors and techniques from others without regard of prestige or social network position, is also optimal when environments are variable and when tasks are simple [59,60,61,62].

Alternatively, trends towards horizontal segmentation may instead reflect a high overlap between the mutual interests of different parts of the organization. Consider that hierarchical organizations sometimes arise when it is necessary to suppress different (and potentially competing) interests of the individuals within organizations. Uniting those individuals under the same cultural framework also allows for functional differentiation [63,64]. In a recent model of the phenomenon, Zefferman [65] examined the relationship between monitoring and punishment for agents in a network and found that non-hierarchical organizations are most likely to arise when the costs of punishment in an organization are high but monitoring is cheap (hierarchical organizations, on the other hand, will arise when punishment is cheap). Under this functional framework, one would expect non-hierarchical organizations to emerge either when interests align and punishment is unnecessary or when punishment is so costly as to prohibit specialization by not allowing managers to focus the tasks of their subordinates.

### 3.3. The Principles of Generative Entrenchment in Organizations

Now that we have laid out the typical pathways and constraints for the development and evolution of organizations, we can consider whether the four major principles of generative entrenchment, originally applied to organismal development, have any bearing on organizational theory. We consider each in turn:

**Principle** **1.** *The proportion and number of mutations that are adaptive declines rapidly at earlier stages in development.*

Within organizations, shifts in the configuration of higher positions should be less common than shifts in lower positions, either with respect to the structural composition of these positions or the individuals on the team. The norms, policies, and institutions present at higher-level positions of an organization should also be more resistant to adaptive changes than those at lower-level positions. That is to say that features at the top of the organization may be “stickier” than features at the bottom of the organization. Relatedly, the number of individuals at the bottom of the organization should far exceed the number of individuals at the top of the organization. Speaking on his involvement in the creation of the Economic Cooperation Administration following the ratification of the Marshall Plan at the end of World War II, Simon [6] notes that the “cell-splitting” process of team recruiting happened most rapidly in non-management positions, while positions reserved for management at the top of the new administration remained frozen. Graeber [66] similarly notes that “optimization” within many corporations of the past generally takes place at the level of lower-level positions than higher-level positions. Graeber specifically argues that this occurs because individuals holding higher-level positions are incentivized against optimizing their roles for efficiency, because doing so might lead to a reduction in their power or influence. We maintain that this mechanism is still phenotypically consistent with our generative entrenchment argument, due to the role of downstream dependencies in the differing malleability of early- versus late-developing structures.

**Principle** **2.** *Evolution should be increasingly conservative at earlier stages of development.*

The generative entrenchment perspective implies that, within organizations, top roles should be similar in their roles and scope, regardless of what the organization is specialized for. This appears to be the case within retail, where companies selling different products nonetheless have nearly identical compositions at the top. Within most major publicly traded companies, we find similar organizational structures at the top of the C-Suite [67]. These regularities exist despite broad rules regulating publicly traded corporations, requiring only that they have a board of directors, not that they possess a C-Suite [36]. Such regularities may be in part the product of business culture within the United States [18,68], but there is not a reason that cultural norms could not be at least partially selected for their role in organizational development. These major components of organizations are generally in place early in the process of development [37].

Wimsatt [21,22] asserts that Principles 1 and 2 hold true because of two additional principles of generative entrenchment, which, packaged together, note that features which are expressed early are likely to be critical for features which appear later.

**Principle** **3.** *Features expressed earlier have a higher probability of being required for features appearing later.*

**Principle** **4.** *Features expressed earlier will on average have a larger number of downstream features dependent on them (are more generatively entrenched).*

From an organizational standpoint, this suggests that organizations should exhibit not just historical, but managerial, dependencies. In other words, not only do early-developing structures scaffold the development of later-developing structures, but those dependencies are also likely to persist even after growth has stabilized. Empirically, Blau [5] notes, structures at the “top” of an organization will tend to be older and have a larger “span of control” than younger components of organizations. The pattern outlined by Principles 3 and 4 therefore suggests that the earliest arising subunits of an organization maintain their relative positions within the organizational structure along with their dependencies through time. In this light, the earliest developing structures in an organization are not only critical for structures which arise later, but they are less likely to be removed from the organization at any given point in its history. Research along this line of reasoning indicates that in times of organization decline, such as during downtrends in sales towards the end of the organizational lifespan, organizations tend to resist reducing administrative size, doing so only as a last resort after subordinate teams had already been cut [69]. This is similar to the parallel case in biology, where radical changes to the appendicular skeleton in vertebrates are more common than radical changes to the (eponymous) spine [70].

## 4. The Evolution of Organizations

Generative entrenchment is, at its heart, an evo-devo theory, that is, a theory of the interplay between evolution and development. Organismal systems are well-suited to this approach, since (1) development can be treated as a product of genetic activation, (2) genes are transmitted to offspring with high fidelity by parents who are endowed with high-fitness traits, and (3) fitness is partly a consequence of successful development. Cultural systems are somewhat more complicated, for a variety of reasons. Individuals transmit information and behavioral practices through a variety of learning mechanisms, such that transmission flows from many sources (not just parents) over an individual’s entire lifespan (not just at conception) [59]. Further, the fidelity of transmission is shaped and constrained by perceptual and communication systems that are themselves shaped by cultural evolution [71,72,73]. This process gets even more complicated when the developmental systems in question are not individuals but organizations.

Organizations that are cohesive may be subject to selection at the group level, whereby those organizations that provide greater overall benefits to their members can outcompete other organizations, thereby ensuring that the structures that are associated with those benefits persist [74]. The framework of “cultural group selection” [75,76,77] has recently been applied to understanding competition between organizations in contexts such as lobster fishing [78], forest management [79], and a large sample of U.S. firms [80]. Despite this success, cultural group selection is predominantly a theory about how intergroup competition promotes the evolution of prosocial norms. It says little about how group structures are transmitted or how they subsequently develop.

Constructing coherent formal theories about the evolution and development of group structures (structures that are nested within larger cultural populations and whose lifespans do not reliably align with the lifespans of individual people) is nontrivial. Many of the core problems in such theory construction are discussed in Smaldino [20]. The presentation of such a theory is beyond the scope of the present discussion. Nevertheless, some speculation is in order. For the purposes of this article, we will gloss over many of the specific details involved in organizational development and evolution, focusing instead on what we see as general principles.

As noted, organismal development evolves through the transmission of successful genetic programs. Organizational development is different. Sometimes, organizations form by splitting off from larger organizations or from the merger of two or more small organizations. Very often, however, organizations form de novo from a mix of need, opportunity, and circumstance. Organizational development is shaped by the decisions of individuals. These decisions are, in turn, shaped by culturally transmitted knowledge and constrained by individual psychology and cultural and environmental factors, such as infrastructure, technology, and economics.

The theory of generative entrenchment is generally consistent with the idea that certain developmental trajectories will be encouraged, through direct or indirect cultural transmission or by encodement in institutional norms, while others will be discouraged. Cultural evolutionary forces should favor processes that produce successful organizations and select against processes that do not. The theory of generative entrenchment can then help us to better understand how we should expect these processes to manifest, and how variation in these processes will be influenced by the stages of organizational development they most strongly influence.

### Institutionalization and the Transmission of Structure

With generative entrenchment biasing the structure of organizations in specific directions, a remaining challenge is to characterize the cultural means through which organizations obtain the initial structures, which are then elaborated upon by generative entrenchment. It is clearly not the case that all companies begin with one small group that then grows large, i.e., a university does not typically start with a chancellor overseeing zero faculty. Put another way, what are the germ organizational structures, and how are they constrained? While most organizations start small, do they all start flat?

Comparative research on organizations indicates this is not likely the case. For example, Lee [17] found a temporal dimension to organizational structures within the video game industry, wherein newly-formed game design organizations have become more hierarchical since the 1990s. This may be due to the fact that these new organizations are increasingly situated in an ecosystem of older and larger competitors. Unless an organization is founded in a completely novel industry, successful templates exist. In fact, it is commonly the case that once a specific organizational niche is identified, the structure of organizations within that niche become institutionalized [68]. That is to say, niche-specific structures become stereotyped and are therefore either required by government, encouraged by niche-specific norms, or, perhaps most commonly, mimicked by other organizations [81]. Additionally, within niches, structures are maintained by expectations set not only by founders, but also employees. A challenge faced by an organization attempting a novel structure is that potential employees may already be enculturated to expect more typical structures and thus to inhabit correspondingly typical roles [18]. Even if that hurdle is overcome, potential employees may balk at atypical roles if they do not transfer laterally for jobs at organizations in similar sectors in the future. Such findings indicate that the expectations of what an ideal organization should look like are held not exclusively in the minds of founders at conception, but also by potential employees.

The copying of successful organizations has several implications for both organizational structure and survival. Consider, for example, the widespread adoption of the open office plan. Despite numerous empirical studies providing evidence that open office plans are disastrous for both worker satisfaction and productivity, such layouts still persist [82,83,84]. Why? As explicitly noted by one workplace analytics consultant, “It is because this is what the workplaces look like at a couple of highly successful tech companies”, referring to companies like Facebook and Google [85]. The biased copying of perceived “optimal” forms of organizations by others is understood to be a form of prestige bias, in which the characteristics of successful models are copied because of their association with success, even in domains in which those characteristics cannot be causally linked with success [86,87].

Organizations may additionally receive other organizational structures and norms by a process of the whole-cloth transmission of other organizations’ structures. Consider, for example, the process of corporate mergers or the acquisition of smaller organizations, which are subsumed under a larger corporate umbrella. By hiring or obtaining a pre-organized unit of another organization, organizations acquire the “local” adaptations that these other organizations possess. Many organizational relationships resemble symbiosis between organisms, in which one or both entities solve problems through their relationship with the other. Furthermore, just as the nature of symbiotic relationships between species varies greatly among biota, relationships between organizations can take on many forms, from obligate to facultative, from mutually beneficial to parasitic. When a smaller organization is fully absorbed by a larger organization, the process may be seen as analogous to organismal symbiogenesis, exemplified by the acquisition of prokaryotic protomitochondria by eukaryotic cells 1.45 billion years ago [88].

Whole-organizational-level knowledge is rarely possessed by any single employee. Indeed, in a study of organizational knowledge among employees, Huising [89] found that even CEOs possessed limited knowledge of their organization’s structure. This is likely to happen as organizations grow to the extent where the top leadership no longer has direct contact with all of their subordinates. As noted by one team member confused by the structure of their company, “The problem is that it was not designed in the first place.” In the case of group-level organizational evolution, transmission takes place via the process of institutionalization in the mind of founders and employees. Besides institutionalization, transmission may also occur through the transfer of tacit knowledge, which is difficult for even the most thoughtful teachers to clearly articulate [90,91]. As such, cultural norms and institutions likely scaffold organizational development in complex ways, which are not well captured by traditional models of cultural evolution [24].

As the product of an evolutionary process, the critical interaction between mutation and selection in organizations take place via the process of generative entrenchment. Organizational development involves the interplay between several forces, including the initial structural seeding by founders, the scaffolding of structural similarities by the expectations of potential employees, and the subsequent elaboration via generative entrenchment. Critically, organizational forms are subject to forces of selection no individual member may themselves be subject to, since the life of an organization is separate from the life of its constituents and even its founders. The link between the selective forces shaping structure and the selective forces shaping agents within the structure requires increased attention within both the organizational and theoretical cultural evolution literature. Furthermore, in both cases, the importance of the relationship between evolution and development cannot be overstated.

## 5. Conclusions

The use of generative entrenchment in biology links two often-disparate perspectives on organismal traits: intra-organismal development and cross-organismal regularities, typically covered under the banners of ontogeny (development) and phylogeny (evolutionary history). By considering the ontogenetic dynamics that produce functional regularities within systems, generative entrenchment provides a framework through which we can understand how early forms of organismal development scaffold the development of later-developing structures, produce complex decision-making architectures, and ensure the conservation of baseline features that are critical for the evolutionary viability of the mature organism.

Similarly, the study of organizations examines the development of self-assembling entities, their optimal structural arrangements, and their survival. While much has been written on the “lifespan” of organizations, few stable theoretical frameworks have been formulated which link the process of organizational development and organizational structure. The theory of generative entrenchment is a potentially fruitful theoretical framework, which explicitly considers this linkage. We have shown how it can help to explain the processes through which organizations take their specific structures, how organizations become adapted to their specific niches, and why organizations exhibit widespread commonalities regardless of which socioeconomic niche they occupy.

At its core, generative entrenchment is a theory about hierarchical path dependency: structures that are composed earliest in organismal development affect the formation of structures that come later (being, by consequence, built upon these earlier structures). The entrenchment of those earliest -developing structures constrains the variation and subsequent evolution of organisms and, we argue, organizations. This theoretical framework provides us with insights for organizational formation, which can be explored empirically and elaborated by future modeling efforts to help us to better understand factors such as the development of hierarchy, the emergence of specialization, and the role of cultural knowledge in how organizations assemble collectively and, ultimately, evolve.

## Data Availability

Not applicable.
